# Maternal dietary diversity and associated factors among pregnant women visiting public health institutions for antenatal care in Addis Ababa, Ethiopia

**DOI:** 10.3389/fgwh.2026.1552532

**Published:** 2026-04-02

**Authors:** Firew Wubshet Birhanu, Dereje Bayissa Demissie

**Affiliations:** 1 Department of Public Health, Sante Medical College, and Menelik II Comprehensive Specialized Hospital, Addis Ababa, Ethiopia; 2 School of Nursing, St. Paul's Hospital Millennium Medical College, Addis Ababa, Ethiopia

**Keywords:** antenatal care, dietary diversity, maternal nutrition, nutrition, pregnant women

## Abstract

**Background:**

Pregnancy is a critical stage with major physiological and biochemical changes, increasing nutritional needs for maternal and fetal growth. These demands make women highly vulnerable to malnutrition. Dietary practices observable eating behaviors are key to meeting these needs and are classified as good or poor. Compared to other life stages, pregnancy carries a higher risk of deficiencies influenced by eating habits.

**Objective:**

This study aimed to assess maternal dietary practices and identify factors associated with good dietary diversity among pregnant women attending public health institutions in Addis Ababa, Ethiopia, in 2024.

**Methods:**

An institution-based cross-sectional study was conducted among 333 pregnant women attending ANC in Addis Ababa from April 24 to May 23, 2024. Data were collected using interviewer-administered questionnaires and a 24-hour dietary recall. Analysis was performed in SPSS version 27, applying descriptive statistics and bivariate logistic regression to identify candidate variables, followed by multivariable logistic regression to determine independent predictors of dietary practice.

**Result:**

The mean age of respondents was 27.91 years (SD ± 4.74). The overall prevalence of good dietary practice was 39% (95% CI: 38.5–39.4), indicating that less than half of the participants met the minimum dietary diversity standard. Multivariable logistic regression identified several significant predictors of good dietary practice. Women without a habit of eating snacks were 72% less likely to achieve good dietary diversity (AOR = 0.28; 95% CI: 0.09–0.81). Conversely, consumption of specific food groups strongly increased the likelihood of good dietary practice: Pulses: AOR = 6.59 (95% CI: 3.36–12.92), Nuts and seeds: AOR = 34.24 (95% CI: 5.23–223.94) and other fruits: AOR = 33.14 (95% CI: 8.74–125.64). These findings underscore the critical role of nutrient-rich foods in achieving dietary diversity. Additionally, 75.2% of participants reported consuming protein-rich foods and fresh fruits within the previous 24 h, suggesting some positive dietary behaviors despite overall low diversity.

**Conclusion and recommendation:**

This study found that the prevalence of good dietary practice among pregnant women was moderate, with several factors significantly influencing outcomes. Positive predictors included husband's occupation (government employment), food cravings, snack consumption, and intake of protein-rich foods, pulses, and meat, poultry, and fish. Conversely, meal skipping during pregnancy was associated with reduced odds of good dietary practice. Policymakers and health planners should strengthen routine screening and counseling on maternal dietary practices during ANC visits. Nutrition education programs should emphasize the importance of diverse food consumption and discourage meal skipping to improve maternal and fetal health outcomes.

## Background

Global prevalence of inadequate dietary diversity among pregnant women remains alarmingly high, with recent data indicating widespread nutritional gaps that threaten maternal and foetal health. While comprehensive global aggregates specifically for pregnant women are limited, proxy data from women of reproductive age (15–49 years) shows only 65% achieving minimum dietary diversity between 2019 and 2023, implying 35% inadequacy, with even lower rates in high-burden regions like Sub-Saharan Africa at 56% inadequate (1, 2). Among pregnant women, country-specific and regional studies from 2024 to 2025 reveal rates exceeding 90% in parts of Africa, underscoring the urgency for targeted interventions (3). Everyone requires an appropriate amount of nutrients for their body systems to function effectively. Adequate nutrition and optimal nutrition are crucial for survival, physical development, cognitive advancement, performance and efficiency, health and well-being (4).

However, nutritional requirements differ with physiological changes such as age, sex, and pregnancy (5). During pregnancy, a woman's body experiences structural, physiological, and biochemical alterations. Biological changes enhance women's nutritional demands. Therefore, pregnant mothers should consume a variety of foods that supply sufficient energy, protein, vitamins, minerals, and hydration (6). Pregnancy represents the most nutritionally demanding period in every woman's life. Pregnant mothers have to make sure they are obtaining adequate nutrients to store energy for their growing bodies and new tissues. With all the stuff happening with their breast and uterus, and the extra energy required to make new tissues, they can be more likely to be exposed to malnutrition (7). Effectively consumption of vital macro- and micro-nutrients in the period of pregnancy helps the health of pregnant women and their babies. Besides, women's health status and the cycle of malnutrition are greatly influenced by the promotion of women's health and the prevention of health care from conception to adulthood. Pregnancy is the period of life when poor dietary practices are prevalent due to a poor dietary pattern, and these poor practices increase the likelihood of intrauterine growth restriction, low birth weight, preterm birth, anemia, increased infection, congenital disability, pre-eclampsia, prenatal, and infant mortality and morbidity (8).

A recent systematic review and meta-analysis revealed that inadequate dietary diversity remains a critical challenge among pregnant women in Sub-Saharan Africa. Prevalence rates exceed 94% in countries such as Burkina Faso, Ghana, Kenya, and Tanzania, with marked urban–rural disparities (38% vs. 62%). In Somalia, inadequacy among antenatal care attendees ranges between 48% and 52%, while certain vulnerable groups in other regions achieve as little as 7% adequacy (2). Key drivers of poor dietary diversity include low utilization of antenatal care services and poor household wealth status, which increase the odds of inadequacy by 1.2–3 times. Additional contributing factors such as food insecurity, limited maternal education, and broader socioeconomic disparities further exacerbate the problem, underscoring the need for targeted interventions to improve maternal nutrition during pregnancy (2, 3). A primary factor in the deaths and disabilities of 3.5 million mothers worldwide is under-nutrition in low- and middle-income countries. This is because under-nutrition throughout the early stages of a child's life can have disastrous physical and psychological implications. Preterm birth, low birth weight (LBW), and unsuccessful birth outcomes are associated with chronic energy shortage, which has been shown in undernourished pregnant women (9).

Maternal diet counseling and social behavior change strategies should be integrated into community-level initiatives, such as health extension programs and engagement of male and female community leaders (10).Nutrition is central to achieving the UN Sustainable Development Goals (SDGs), directly influencing 12 of the 17 goals, while the remaining five support nutritional improvements. Strengthening maternal nutrition is therefore critical for global health and sustainable development (1).

Improving maternal and child nutrition is a national priority in Ethiopia, as outlined in the Health Sector Transformation Plan and the National Nutrition Program II (NNP-II), which aims to enhance the nutritional status of adolescent girls (10–19 years) and women (15–49 years) (11). Despite these strategic commitments, evidence shows that nutrition education during antenatal care (ANC) remains inadequate, largely due to systemic and operational barriers (10). This gap is critical because maternal dietary diversity is a key determinant of maternal and fetal health, influencing pregnancy outcomes and long-term well-being. Urban centers like Addis Ababa present unique opportunities and challenges for maternal nutrition, given their diverse food environments and socio-economic disparities. However, limited data exist on dietary practices and associated factors among pregnant women in these settings. Understanding these patterns is essential for designing context-specific interventions, strengthening ANC-based nutrition counseling, and informing policy decisions aligned with Ethiopia's national nutrition goals and the Sustainable Development Goals (SDGs). Therefore, this study was undertaken to assess maternal dietary practices and identify associated factors among pregnant women attending public health institutions in Addis Ababa, providing evidence to guide nutrition-sensitive policies, health programs, and future research.

## Methods

### Study setting, design, and population

This study was conducted in Addis Ababa, the capital city of Ethiopia, which serves as the country's political and economic hub. According to the Central Statistics Agency (2023), Addis Ababa has an estimated population of 5.46 million, comprising 2.84 million women and 2.62 million men, reflecting a 4.46% increase from 2022. The city spans approximately 526.99 km^2^ with a population density of 5,535.8 persons per km^2^. Notably, Ethiopia's adolescent and youth population is rapidly growing, accounting for 33.8% of the national population.

Administratively, Addis Ababa operates under a three-tier system: the city government, 11 sub-city administrations, and 99 kebele administrations, encompassing 116 woredas. The city's health infrastructure includes seven hospitals and 97 health centers under the city administration, alongside five specialized referral hospitals, one Defense Forces referral hospital, and one Federal Police hospital managed by federal institutions. The health workforce comprises approximately 12,940 healthcare professionals.

The study employed a multi-center, institution-based cross-sectional design and was conducted from April 24 to May 23, 2024. The target population consisted of pregnant women attending antenatal care (ANC) services in public health facilities across Addis Ababa. Participants were selected using systematic random sampling. Inclusion criteria were pregnant women residing in Addis Ababa for at least six months and free from severe medical conditions. Exclusion criteria included women who were critically ill, unable to communicate, had physical or language limitations, mental disorders, or were unable to provide verbal consent. Sample size determination and Sampling procedure.

The sample size was determined using the single population proportion formula, based on an estimated prevalence of good dietary practice during pregnancy of 27.2% (12), a 5% margin of error, and a 95% confidence level. This calculation yielded a sample size of 303 participants. After accounting for a 10% non-response rate, the final sample size was 333 pregnant women.

### Sampling procedure

The study employed a systematic random sampling technique across selected public health institutions in four randomly chosen sub-cities of Addis Ababa: Akaki Kality, Lideta, Kirkos, and Yeka. These sub-cities represent approximately 30% of the city's 11 sub-cities. Health facilities included Gelan Health Center, Ethiopian Federal Police Referral Hospital, Armed Forces Comprehensive Specialized Hospital, Kazanchis Health Center, and Menelik II Comprehensive Specialized Hospital. Proportional allocation was applied based on the average monthly ANC attendance at each facility, calculated from the previous three months’ records. The total monthly ANC attendance across these institutions was 306, 978, 225, 122, and 483, respectively (8).

**Figure 1:**
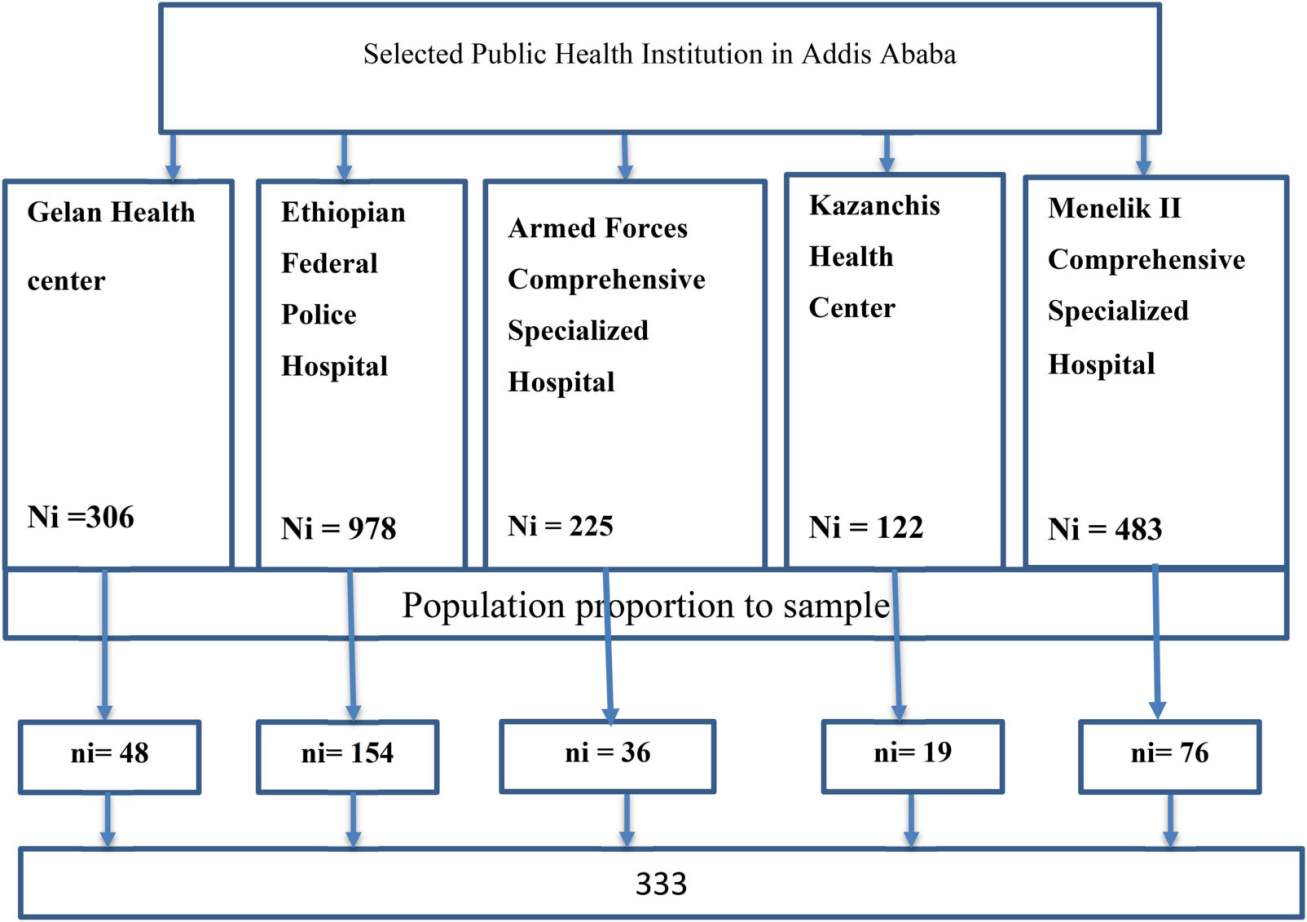
Schematic presentation of sampling procedure pregnant women visiting public health institutions in Addis Ababa for antenatal care, Addis Ababa, Ethiopia, 2024.

The sampling interval (k) was computed as 6 (2,114 ÷ 333), using the expected number of ANC attendees during the study period and the required sample size. The first participant was selected randomly by lottery, and subsequent participants were chosen at every sixth interval using a systematic approach. See details in Figure 1 schematic presentation.

### Operational definitions

Antenatal Care (ANC): A preventive health service designed to promote maternal and fetal well-being during pregnancy. It involves routine check-ups that enable healthcare providers to monitor progress, manage potential complications, and encourage healthy lifestyle practices beneficial to both mother and child.

Maternal Nutrition: The process of ensuring adequate nutrient intake and dietary planning before, during, and after pregnancy, including supplementation and balanced diet strategies to support maternal and fetal health.

Poor Dietary Diversity Score: Defined as the consumption of fewer than five food groups out of the ten recommended groups within the 24 h preceding the survey. This is coded as 0 (13).

Good Dietary Diversity Score: Defined as the consumption of five or more food groups out of the ten recommended groups within the 24 h preceding the survey. This is coded as 1 (13).

Knowledge: Refers to the understanding of nutrition concepts and practices acquired through learning and experience during pregnancy.

### Data collection and quality assurance

A structured questionnaire and the 24-hour recall method were used to collect the data which is adapted from the Food and Agriculture Organization's (FAO) Guidelines for measuring dietary diversity in households and individuals (FAO, 2016) and (Exploring the new indicator Minimum Dietary Diversity-Women results from Burkina Faso 2015). Ten food groups in total were considered in this study: cereals (grains, white roots and tubers, and plantains), pulses (beans, peas, and lentils), nuts and seeds, dairy products, meat, poultry, and fish, eggs, dark green leafy vegetables, other vitamin A-rich fruits and vegetables, other vegetable and other fruits (14).

Dietary diversity among pregnant women was assessed retrospectively using a 24-hour food intake checklist, where participants indicated consumption of food groups with Yes/No responses to calculate the dietary diversity score.

A structured questionnaire (Supplementary Appendix A1) was developed in English and translated into Amharic, Ethiopia's national language. Data collectors received two days of training on study objectives, questionnaire content, confidentiality, and respondent rights. A pretest was conducted on 5% of the sample at a health facility outside the study area, and the tool was refined based on feedback.

Data collection was supervised daily by the principal investigator to ensure quality, completeness, and consistency, with immediate corrections applied when necessary. Interviews were conducted as exit interviews by trained midwives, nurses, and public health professionals from non-study facilities. Prior to each interview, verbal consent was obtained, and participants’ ID numbers were recorded to track missing or misplaced data.

### Data management and analysis

Data were collected using Kobo Toolbox (version 2023.2.4), cleaned, and exported to SPSS version 27 for analysis. Variables were defined, categorized, and re-coded before generating descriptive statistics, including frequencies, cross-tabulations, and chi-square tests to compare distributions.

Binary logistic regression was performed to assess associations between the outcome variable and independent predictors. Variables with a *p*-value < 0.25 in bivariate analysis were included in multivariable logistic regression (enter method) to control for potential confounders and identify significant predictors.

The strength of associations was expressed using Crude Odds Ratios (COR) and Adjusted Odds Ratios (AOR) with 95% confidence intervals (CI). Model fitness was evaluated using the Hosmer–Lemeshow goodness-of-fit test, with an insignificant result (*p* > 0.05) indicating adequate fit. Statistical significance was declared at *p* < 0.05.

## Result

### Socio-demographic characteristics of the study participant

A total of 326 pregnant women participated in the study, yielding a 97.9% response rate (7 participants did not complete the questionnaire). The mean age of respondents was 27.85 years (SD ± 4.73), with the majority (65.6%) aged 25–34 years, indicating that most participants were in their prime reproductive years. Nearly all participants were married (96.0%), reflecting the cultural norm of marriage during pregnancy in this setting.

In terms of education, 39.6% had completed secondary school, suggesting moderate educational attainment among the sample. Regarding occupation, 39.0% were government employees, highlighting a significant proportion of women engaged in formal employment.

Household composition showed that 51.5% lived in families with 3–4 members, which may influence resource allocation and dietary diversity.

Additionally, 37.7% reported a monthly household income above 9,000 Ethiopian birr, indicating that a substantial proportion of participants belonged to relatively higher-income households compared to national averages. These characteristics provide important context for understanding maternal nutrition behaviors and associated factors in Addis Ababa (Table 1).

**Table 1:** Distribution of socio-demographic characteristics of study participant in Addis Ababa public health institutions, in 2024 (*n* = 326).

Variables	Category	Frequency (*n*)	Percentage %
Age	15–24	79	24.2
	25–34	214	65.6
	≥35	33	10.1
Marital status	Single	11	3.4
	Married	313	96.0
	Separated/widowed	2	0.6
Educational level	Has no formal education	25	7.6
	Primary education (1–8)	68	20.9
	Secondary education (9–12)	129	39.6
	College and above	104	31.9
Husband's educational level	Has no formal education	4	1.2
	Primary education (1–8)	44	13.5
	Secondary education (9–12)	108	33.1
	College and above	157	48.2
Occupation	House wife	110	33.7
	Government employee	127	39.0
	Business	5	1.5
	Laborer	7	2.1
	Student	8	2.5
	Self employed	62	19.0
	Other^a^	7	2.1
Husband's occupation	Government employee	182	54.65
	Business	22	6.61
	Laborer	19	5.71
	Self employed	87	26.13
	Other^b^	10	3
Family's monthly income in ETB 1USD = 125 Birr	<3,000	29	8.9
	3,000–6,000	77	23.6
	6,000–9,000	97	29.8
	>9,000	123	37.7
Family size	1–2	112	34.4
	3–4	168	51.5
	>4	46	14.1

^a^ Denotes housemaid and unemployed.

^b^ Denotes student, farmer and unemployed.

### Pregnancy and obstetric related characteristics of the respondent

Among the 326 pregnant women surveyed, 65.6% (*n* = 214) had a previous pregnancy. Of these, 74.8% reported ≤2 previous pregnancies, while 25.2% had three or more.

Regarding childbirth history, 62.0% (*n* = 202) had given birth before, and almost all (98.0%) delivered their last baby in a health facility, with only 1.98% delivering at home. Among those with prior births, 6.4% reported a history of child death, and 38.4% of these losses occurred after the child was older than one year.

Antenatal care (ANC) utilization was high, with 88.0% (*n* = 287) attending ANC during the current pregnancy. Of these, 75% had ≤4 visits, while 25% attended ≥4 visits. Most women (74.2%) initiated ANC in the first trimester, while 24.4% started in the second and 1.4% in the third trimester. At the time of the survey, 50.3% were in their first trimester, 36.5% in the second, and 13.2% in the third. Among women with previous pregnancies, 27.6% reported a history of miscarriage, and 88.1% of these occurred only once (Table 2).

**Table 2:** Previous pregnancy and obstetric related status of the respondents in Addis Ababa public health institutions in 2024 (*n* = 326).

Variables	Category	Frequency	Percent
You have ever got pregnant before (*n* = 326)	Yes	214	65.6
	No	112	34.4
Number of previous pregnancies (*n* = 214)	≤2	160	74.77
	>=3	54	25.23
You have ever given birth before (*n* = 214)	Yes	202	94.4
	No	12	5.6
Number of times you gave birth before (*n* = 202)	≤2	164	81.2
	>=3	38	18.8
Pregnancy interval b/n the past and current pregnancy in years (*n* = 214)	<2 years	38	17.8
	2–4 years	101	47.2
	4–6 years	46	21.5
	>6 years	29	13.5
You have ever got child death before (*n* = 202)	Yes	13	6.4
	No	189	93.6
Your last baby is died in the age of (*n* = 13)	Within 1 month	4	30.8
	b/n 1 month and 1 year	4	30.8
	>1 year	5	38.4
The delivery place of the last child (*n* = 202)	Health institution	198	98.02
	Home	4	1.98
Number of live children (*n* = 202)	≤2	166	82.2
	>=3	36	17.8
Miscarriages have occurred before (*n* = 214)	Yes	59	27.6
	No	155	72.4
Number of times miscarriage has occurred (*n* = 59)	1 time	52	88.1
	2 times	7	11.9
Sick during previous pregnancy (*n* = 214)	Yes	61	28.5
	No	153	71.5
ANC follow up (*n* = 326)	Yes	287	88.0
	No	39	12.0
Number of visits for ANC follow up (*n* = 287)	≤4 visits	215	75
	>4 visits	72	25
Time of initiation of ANC follow up (*n* = 287)	1st trimester	213	74.2
	2nd trimester	70	24.4
	3rd trimester	4	1.4
Stage of pregnancy (*n* = 326)	1st trimester	43	13.2
	2nd trimester	119	36.5
	3rd trimester	164	50.3

### Food group categories based on 24-hour recall

The 24-hour dietary diversity of pregnant women was assessed using the Minimum Dietary Diversity for Women (MDD-W) indicator, where a woman is considered to have achieved adequate diversity if she consumed at least five out of ten food groups in the previous 24 h.

Analysis revealed that 95.1% of participants consumed cereals, grains, white roots, tubers, and plantains, indicating heavy reliance on staple foods. Intake of pulses (beans, peas, lentils) was reported by 31.0%, while only 8.3% consumed nuts and seeds, reflecting limited diversity in plant-based protein sources. Among animal-source foods, 41.4% consumed milk and milk products, 25.5% consumed meat, poultry, or fish, and 32.8% consumed eggs.

Regarding fruits and vegetables, 40.5% consumed dark green leafy vegetables, 15.6% consumed other fruits, and 91.7% consumed other vegetables. Additionally, 44.2% reported intake of vitamin A-rich fruits and vegetables. These findings highlight a strong dependence on staple foods and low consumption of nutrient-dense food groups, underscoring the need for targeted interventions to improve dietary diversity among pregnant women (Table 3).

**Table 3:** Food group category of 24-hour recall of pregnant women's dietary practice of the respondents in Addis Ababa public health institutions in 2024 (*n* = 326).

Food group	Response	Frequency	Percent
Cereals/grains, white roots tubers and plantain	Yes	310	95.1
	No	16	4.9
Pulses (beans, peas and lentils)	Yes	101	31.0
	No	225	69.0
Nuts and seeds	Yes	27	8.3
	No	299	91.7
Dairy products	Yes	135	41.4
	No	191	58.6
Meat, poultry and fish	Yes	83	25.5
	No	243	74.5
Eggs	Yes	107	32.8
	No	219	67.2
Dark green leafy vegetables	Yes	135	40.5
	No	194	59.5
Other vitamin A rich fruits and vegetables	Yes	144	44.2
	No	182	55.4
Other vegetable	Yes	299	91.7
	No	27	8.3
Other fruits	Yes	51	15.6
	No	275	84.4

**Figure 2:**
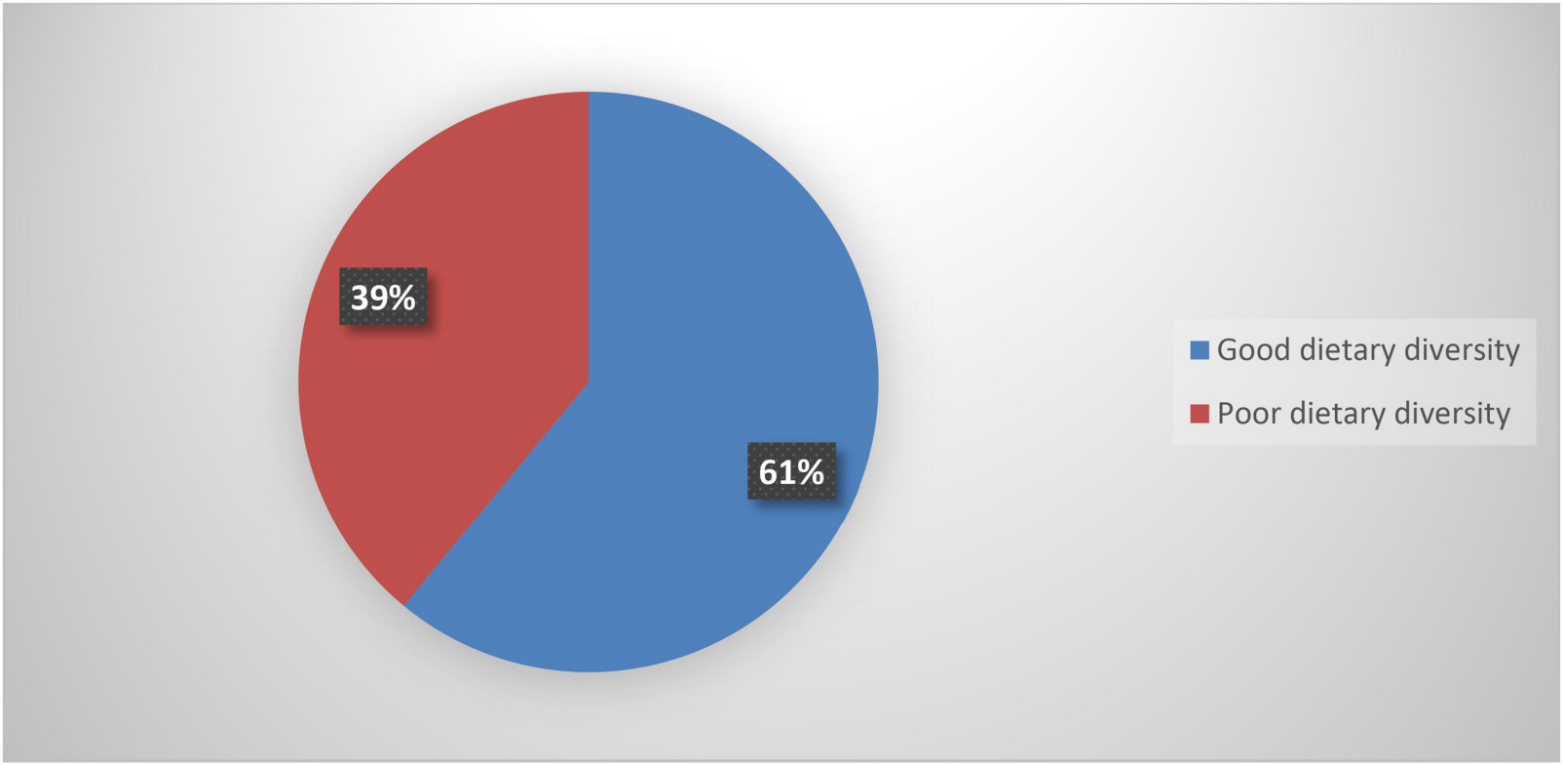
Dietary diversity of pregnant women attending ante natal care at public health institutions in Addis Ababa, Ethiopia, 2024 (*n* = 326).

This study determined that the overall magnitude of good dietary practice among pregnant women was 39% (95% CI: 38.04, 39.96) Figure 2.

### Factors associated with dietary practice among pregnant women

Bivariate logistic regression statistical test was done and the presence of any relationship between independent variable with dietary practice dependent variable among pregnant mothers who seek reproductive health service in Addis Ababa health facilities was done. Factors like occupation status of husband, craving of food not normally consumed, avoidance of some food items during current pregnancy, habit of eating snack, number of meals per day and eating consumption of protein rich foods during reveal statistically significant association (*p* < 0.02) with dietary practice. Age, marital status, educational status of mothers, education status of husband, family monthly income and family, size has no association by bivariate regression model.

### Predictors of good dietary practice among pregnant women

To identify independent predictors of dietary practice, variables with *p* < 0.05 in bivariate logistic regression were included in the multivariable model. Only variables that remained significant at *p* < 0.05 were considered true predictors. The analysis revealed that snack-eating habits and consumption of specific food groups’ pulses, nuts and seeds, dairy products, meat/poultry/fish, eggs, dark green leafy vegetables, and fruits were significantly associated with good dietary diversity.

Pregnant women without a habit of eating snacks were 72% less likely to achieve good dietary diversity compared to those who did (AOR = 0.28; 95% CI: 0.09–0.81). Conversely, consumption of nutrient-rich food groups strongly predicted good dietary practice. Women who consumed pulses were 6.6 times more likely to meet dietary diversity standards (AOR = 6.59; 95% CI: 3.36–12.92). Intake of nuts and seeds showed the strongest association, with women being 34 times more likely to achieve good diversity (AOR = 34.24; 95% CI: 5.23–223.94) (Table 4). Similarly, consumption of dairy products (AOR = 15.92; 95% CI: 6.71–37.75), meat/poultry/fish (AOR = 12.53; 95% CI: 4.77–32.91), and eggs (AOR = 7.02; 95% CI: 4.20–11.74) significantly increased the odds of good dietary practice.

**Table 4:** Binary logistic regression analyses showing factors associated with maternal dietary practice among pregnant women in Addis Ababa public health institutions, Addis Ababa, Ethiopia 2024 (*n* = 326).

Variables	Category	Dietary diversity		COR	PV
		Good no (%)	Poor no (%)		
Husband's occupation status	Business	9 (7.1%)	13 (6.5%)	0.65 (0.25–1.70)	0.381
	Government employee	83 (65.4%)	92 (46.2%)	**0.49 (0.29–0.85)**	**0.012**
	Laborer	2 (1.6%)	17 (8.5%)	3.82 (0.83–17.74)	0.087
	Other (Farmer, Student and Unemployed)	1 (0.8%)	9 (4.5%)	4.05 (0.48–33.5)	0.195
	Self employed	27 (21.3%)	60 (30.2)	1	
Crave food not normally consumed	No	84 (66.1%)	166 (83.4%)	**2.57 (1.52–4.35)**	**0.000**
	Yes	43 (33.9%)	33 (16.6%)	**1**	
Avoidance of some food items during current pregnancy because of personal dislike	No	2.2 (55.0%)	19 (31.1%)	**2.7 (1.18–6.17)**	**0.018**
	Yes	18 (45.0%)	42 (68.9%)	1	
Avoidance of some food items during current pregnancy because of culture, illness& fear of parasite	Yes	21 (52.5%)	16 (26.2%)	**3.1 (1.34–7.22)**	**0.008**
	No	19 (47.5%)	45 (73.8%)	**1**	
Having a habit of eating snack	Yes	67 (52.8%)	76 (38.2)	**1.80 (1.15–2.84)**	**0.010**
	No	60 (47.2%)	123 (61.8%)	1	
Number of meals per day	1–2	4 (3.1%)	4 (2.0%)	1.08 (0.252–4.675)	0.912
	3–4	85 (66.9%)	16 (80.4%)	2.04 (1.204–3.469)	0.008
	≥5	38 (29.9%)	35 (17.6%)	1	
Eating protein rich foods during pregnancy	Yes	81 (63.8%)	160 (80.4%)	**0.429 (0.259–0.710)**	**0.000**
	No	46(36.2%)	39(19.6%)	1	

Vegetable and fruit intake also played a critical role. Women who consumed dark green leafy vegetables were nearly 10 times more likely to achieve good dietary diversity (AOR = 9.82; 95% CI: 5.18–18.62), while those who ate other fruits had 33 times higher odds (AOR = 33.14; 95% CI: 8.74–125.64). Notably, socio-demographic factors such as husband's occupation, pregnancy interval, food cravings, and meal skipping were not significant predictors in the multivariable model (Table 5).

**Table 5:** Multivariable logistic regression analyses showed factors associated with maternal dietary practice among pregnant women in Addis Ababa public health institutions, Addis Ababa, Ethiopia 2024 (*n* = 326).

Variables	Category	Dietary practice		COR	AOR	*P*- value
		Good no (%)	Poor no (%)			
Do you a have a habit of eating snack?	No	67 (52.8%)	76 (38.2%)	**1.80 (1.15–2.83)**	**0.28 (0.09- 0.81)**	**0**.**000**
	Yes	60 (47.2%)	123 (61.8%)	1		
Consumed Pulses (beans, peas and lentils) within 24 h?	Yes	65 (51.2%)	36 (18.1%)	**4.74 (2.87–7.83)**	**6.59 (3.36- 12.92)**	**0**.**000**
	No	62 (48.8%)	163 (81.9%)	1	1	
Consumed Nuts and seeds within 24 h?	Yes	21 (16.5%)	6 (3.0%)	**6.37 (2.49–16.27)**	**34.24 (5.23- 223.94)**	**0**.**000**
	No	106 (835%)	193 (970%)	1	1	
Consumed dairy products	Yes	83 (65.4%)	52 (26.1%)	5**.**33 (3**.**28–8**.**64)	**15.92 (6.71- 37.75)**	**0**.**000**
	No	44 (34.6%)	147 (73.9%)	1	1	
Consumed meat, poultry and fish within 24 h?	Yes	80 (63.0%)	163 (81.9%)	**2.66 (1.59–4.42)**	**12.53 (4.77- 32.91)**	**0**.**000**
	No	47 (37.0%)	36 (18.1%)	1	1	
Consumed eggs within 24 h?	Yes	74 (58.3%)	33 (16.6%)	**5.4 (3.15–8.27)**	**7.02 (4.20- 11.74)**	**0**.**000**
	No	53 (41.7%)	166 (83.4%)	1	1	
Consumed dark green leafy vegetables within 24 h	Yes	81 (63.8%)	51 (25.6%)	**5.11 (3.15–8.27)**	**9.82 (5.18- 18.62)**	**0**.**000**
	No	46 (36.2%)	148 (74.4%)	1	**1**	
Consumed other fruits including wild fruits and 100% fruit juice made from these within 24 h?	Yes	88 (69.3%)	187 (94.0%)	**6.90 (3.44–13.83)**	**33.14 (8.74- 125.64)**	**0**.**000**
	No	39 (30.7%)	12(6.0%)	1	1	

Bold values indicates statistically significant at *P* < 0.05.

## Discussion

This study assessed dietary practices and their determinants among pregnant women attending public health facilities in Addis Ababa. The prevalence of good dietary practice was 39%, indicating a moderate level of dietary diversity. Women who consumed nutrient-dense foods such as nuts, fruits, dairy, pulses, and animal-source foods were significantly more likely to achieve good dietary diversity, while those who skipped snacks were less likely. These findings emphasize the need for targeted interventions promoting frequent, varied meals and access to diverse, nutrient-rich foods during pregnancy. This magnitude is higher than the study conducted in Liberia (16.5%) (15), West Gojam zone, North-west Ethiopia (19.9%) (13), Mizan Aman south west Ethiopia (29.71%) (8), Farta district South Gondar zone north west Ethiopia (27.2%) (12) Bench-Sheko and Kaffa Zones, Southwest Ethiopia (23.7) (8), Gedeo zone, southern Ethiopia (32.2%) (16) Several contextual factors may explain this difference. As an urban center, Addis Ababa offers notable advantages, including improved access to diverse food markets, better health infrastructure, and more frequent exposure to nutrition messaging during antenatal care visits. Socioeconomic factors such as higher household income, greater employment opportunities, and easier availability of nutrient-rich foods may also contribute to the relatively better dietary diversity observed. However, despite improved socioeconomic conditions, many pregnant women reported insufficient time to feed themselves adequately. This paradox may reflect the realities of urban life. Pregnant women in Addis Ababa often experience high workloads, balancing formal employment with extensive domestic responsibilities. Additionally, cultural norms may limit women's autonomy over food preparation and meal timing, further constraining their ability to prioritise their own nutritional needs. These factors contribute to time poverty, a well-documented challenge in urban settings, which can hinder the ability to consume diverse and nutritionally adequate meals even when resources are available. This complexity has now been explicitly incorporated into the discussion.

This study magnitude of good dietary practices in this study is in line with the study done in Bahir Dar town, north-east Ethiopia (39.3%) (7) and this magnitude is slightly lower than the study conducted in Dessie town, North-eastern Ethiopia (45.2%) (6) and still lower than the study conducted in Akaki Kality sub city Addis Ababa, Ethiopia 49.3% (17), Kolfe Keranio sub-city, Addis Ababa, Ethiopia (60.9%) (18) and the Western hill region of Nepal (55.1%) (19). Urban sub-cities like Kolfe Keranio may have better food availability and higher socioeconomic status compared to other areas, contributing to higher dietary diversity. Additionally, differences in findings across studies may reflect methodological variations, including differences in sample size, study settings, and timing.

This study found that pregnant women who did not crave foods outside their usual diet were 3.5 times more likely to achieve good dietary diversity. This contrasts with findings from Southern Ethiopia 38 but aligns with results from Misha Woreda (20). The discrepancy may be due to methodological differences or confounding factors, such as variations in cultural food norms or sample characteristics. These inconsistencies may stem from differences in study populations, cultural food norms, or variations in how cravings were measured across studies. Moreover, the physiological demands of early pregnancy particularly nausea and morning sickness—may interact with women's workload and stress levels, influencing both cravings and dietary intake. High workloads, psychological stress, and competing responsibilities can exacerbate nausea, leading to reduced appetite or selective eating patterns that negatively affect dietary diversity. This interplay between physiological symptoms, work-related stress, and pregnancy-related demands helps contextualize the behavioral patterns observed in this study and has now been more clearly articulated in the discussion.

Additionally, 75.1% of participants reported eating three to four meals daily, consistent with findings from Algeria 39, suggesting that regular meal frequency is a common practice in diverse settings. Importantly, women who consumed pulses were 6.5 times more likely to have good dietary diversity, similar to findings from Ambo District, West Shoa Zone (21). This reinforces the role of incorporating multiple food groups’ particularly plant-based proteins in meeting nutritional requirements during pregnancy. Craving unusual foods may reflect dietary instability or limited access to preferred foods, while regular meal patterns and inclusion of pulses indicate better food security and awareness of nutrition. These behaviors likely contribute to improved dietary diversity and overall maternal health.

Low consumption of animal-source foods remains a common challenge in many Ethiopian settings and may be influenced by several contextual factors, including cultural norms, economic constraints, religious fasting practices, and food taboos that restrict the intake of certain foods during pregnancy. In this study, pregnant women who consumed dairy products were 84% more likely to achieve good dietary practice compared to those who did not, a finding consistent with reports from Ambo District and Dire Dawa City (21, 22). Likewise, women who consumed meat, poultry, and fish were 12.5 times more likely to attain good dietary diversity, supporting evidence from similar studies conducted in Ethiopia (21, 22). These associations underscore the critical contribution of animal-source foods to improving dietary diversity and enhancing nutrient adequacy during pregnancy. Dietary diversity is widely used as a proxy indicator for micronutrient sufficiency, which is essential for supporting maternal immunity, fetal development, and overall health. Improved access to and consumption of nutrient-rich foods such as dairy, eggs, and meat may also reflect better household food security, greater market availability, and heightened awareness of maternal nutrition. Collectively, these factors contribute to improved maternal nutrition outcomes and reinforce the importance of promoting culturally appropriate strategies to increase intake of animal-source foods where feasible.

Pregnant women who consumed eggs were seven times more likely to achieve good dietary diversity compared to those who did not, consistent with findings from Dire Dawa City, Eastern Ethiopia (22). Similarly, women who consumed dark green leafy vegetables were 9.8 times more likely to have good dietary practice, aligning with results from Ambo District, West Shoa Zone (22). Moreover, consumption of other fruits increased the likelihood of good dietary practice by 33 times, which is in agreement with studies conducted in Kenya (23), and the Central Rift Valley of Ethiopia (24). These associations highlight the critical role of nutrient-dense foods such as eggs, green leafy vegetables, and fruits in improving maternal dietary diversity. Eggs provide high-quality protein and essential micronutrients, while green leafy vegetables and fruits supply vitamins and minerals vital for fetal growth and maternal health. The strong association with fruit consumption may reflect better access to markets and awareness of nutrition benefits in urban settings. Promoting these food groups during pregnancy can significantly enhance dietary adequacy and reduce risks of malnutrition-related complications. Furthermore the present study found no association among husbands of pregnant women, husband occupational status, pregnancy interval years, craving unusual foods, skipping meals, consuming protein rich foods however in other studies Addis Ababa, Ethiopia (13) were significantly associated with maternal good dietary practices.

### Limitation

This study's cross-sectional design limits the ability to establish causal relationships between independent variables and maternal dietary practices. The use of a 24-hour recall method may introduce recall bias, as participants might have difficulty accurately remembering food intake. Additionally, the wide confidence intervals observed for certain food groups likely reflect sparse data, which we have acknowledged in the discussion and noted as a limitation. Although sensitivity checks were conducted to confirm model stability, caution is warranted when interpreting these associations.

Furthermore, the study did not include important socio-economic and cultural determinants such as household food security, decision-making dynamics, women's empowerment, and cultural food taboos. These factors are critical for understanding dietary behaviors and should be incorporated in future research to provide a more comprehensive analysis.

## Conclusion

This study found that good dietary practice among pregnant women in Addis Ababa was moderate (39%), with significant predictors including snack consumption and intake of nutrient-rich foods such as pulses, nuts and seeds, dairy products, meat, poultry, fish, eggs, dark green leafy vegetables, and fruits. These findings highlight the urgent need to strengthen maternal nutrition strategies during pregnancy.

### Policy and practice implications

Integrate Nutrition Counseling into ANC Services: The Ethiopian Ministry of Health should prioritize strengthening nutrition education and counseling within antenatal care programs and monitor both quality and coverage.

Capacity Building for Health Providers: Train healthcare professionals to deliver effective, culturally appropriate dietary guidance and support pregnant women in adopting diverse diets.

Community Engagement: Mobilize community leaders and local organizations to promote healthy eating behaviors and improve access to nutrient-rich foods.

### Future research

Further studies should explore maternal dietary practices at regional and national levels to provide a comprehensive picture. Prospective cohort studies are recommended to identify causal relationships and specific determinants of dietary diversity. Such evidence will inform tailored interventions and public health strategies aimed at reducing maternal malnutrition and improving pregnancy outcomes.

## Data Availability

The original contributions presented in the study are included in the article/Supplementary Material, further inquiries can be directed to the corresponding author.

## References

[1] Atukunda P Eide WB Kardel KR Iversen PO Westerberg AC. Unlocking the potential for achievement of the UN sustainable development goal 2–‘zero hunger’–in Africa: targets, strategies, synergies and challenges. Food Nutr Res. (2021) 65:10–29219. 10.29219/fnr.v65.7686 PMC8254460 34262413

[2] Zegeye AF Mekonen EG Tamir TT Workneh BS. Prevalence and determinants of inadequate dietary diversity among pregnant women in four Sub-Saharan Africa countries: a multilevel analysis of recent demographic and health surveys from 2021 to 2022. Front Nutr. (2024) 11:1405102. 10.3389/fnut.2024.1405102 39301417 PMC11410580

[3] Martin-Cañavate R Trigo E Romay-Barja M Faria LM Gerardo AS Aguado I Dietary diversity in pregnant women and its association with household food security in rural southern Angola. Matern Child Nutr. (2025) 21(4):e70051. 10.1111/mcn.70051 40457576 PMC12454169

[4] Aliwo S Fentie M Awoke T Gizaw Z. Dietary diversity practice and associated factors among pregnant women in north east Ethiopia. BMC Res Notes. (2019) 12(1):123. 10.1186/s13104-019-4159-6 30845950 PMC6407270

[5] Daba G Beyene F Garoma W Fekadu H. Assessment of nutritional practices of pregnant mothers on maternal nutrition and associated factors in Guto Gida Woreda, east Wollega zone, Ethiopia. Sci Tech Arts Res J. (2013) 2(3):105–13. 10.4314/star.v2i3.98748

[6] Diddana TZ. Factors associated with dietary practice and nutritional status of pregnant women in Dessie town, northeastern Ethiopia: a community-based cross-sectional study. BMC Pregnancy Childbirth. (2019) 19(1):517. 10.1186/s12884-019-2649-0 31870426 PMC6929309

[7] Nana A Zema T. Dietary practices and associated factors during pregnancy in northwestern Ethiopia. BMC Pregnancy Childbirth. (2018) 18(1):183. 10.1186/s12884-018-1822-1 29801471 PMC5970492

[8] Abel G Abebaw M Amare G. Dietary Practice and Associated Factors Among Pregnant Women at Public Health Institution in Mizan-Aman Town, Southwest Ethiopia. (2021).

[9] Hasan MM Ahmed S Soares Magalhaes RJ Fatima Y Biswas T Mamun AA. Double burden of malnutrition among women of reproductive age in 55 low-and middle-income countries: progress achieved and opportunities for meeting the global target. Eur J Clin Nutr. (2022) 76(2):277–87. 10.1038/s41430-021-00945-y 34040202 PMC8152189

[10] Alehegn MA Fanta TK Ayalew AF. Exploring maternal nutrition counseling provided by health professionals during antenatal care follow-up: a qualitative study in Addis Ababa, Ethiopia-2019. BMC Nutr. (2021) 7(1):20. 10.1186/s40795-021-00427-1 34092250 PMC8183076

[11] (FMOH) FDRoE. National Nutrition Program 2016–2020. Addis Ababa: Ministry of Health (2016). p. 88. Available online at: www.unicef.org/ethiopia/National_Nutrition_Programme.pdf (Accessed February 10, 2024).

[12] Belay WS Cherkos EA Taye EB. Dietary practice during pregnancy and associated factors among pregnant women in Farta district, south Gondar zone, northwest Ethiopia, 2021. Clin Epidemiol Glob Health. (2022) 14:100968. 10.1016/j.cegh.2022.100968

[13] Kebede AN Sahile AT Kelile BC. Dietary diversity and associated factors among pregnant women in Addis Ababa, Ethiopia, 2021. Int J Public Health. (2022) 67:1605377. 10.3389/ijph.2022.1605377 36405528 PMC9668879

[14] FAO F. Minimum Diet Diversity for Women: A Guide for Measurement. Rome: Food and Agriculture Organization (2016). Available online at: http://www.fao.org/3/a-i5486e.pdf (Accessed February 10, 2024).

[15] Geetah SS Intiful FD Dogbe YY Pereko K Asante M. Anaemia and dietary diversity among pregnant women in Margibi and Grand Cape mount counties, Liberia. Health Sci Invest J. (2023) 4(2):560–7. 10.46829/hsijournal.2023.12.4.2.560-567

[16] Yalewdeg M Birhane M Adissu Y. Dietary practices and their determinants among pregnant women in Gedeo zone, southern Ethiopia: a community-based cross-sectional study. Nutr Diet Suppl. (2020) 12:267–75. 10.2147/NDS.S267453

[17] Koster Y Niguse B. Dietary practice and associated factors among pregnant women attending ante Natal Care, Akaki Kality Sub City Addis Ababa, Ethiopia 2022. (2023).

[18] Tefera W Brhanie TW Dereje M. Dietary diversity practice and associated factors among pregnant women attending ANC in Kolfe Keranyo sub city health centers, Addis Ababa, Ethiopia. medRxiv. (2020):2020–04. 10.1101/2020.04.27.20081596

[19] Shrestha V Paudel R Sunuwar DR Lyman ALT Manohar S Amatya A. Factors associated with dietary diversity among pregnant women in the western hill region of Nepal: a community based cross-sectional study. PLoS One. (2021) 16(4):e0247085. 10.1371/journal.pone.0247085 33831015 PMC8031299

[20] Abute L Beyamo A Erchafo B Tadesse T Sulamo D Sadoro T. Dietary practice and associated factors among pregnant women in Misha Woreda, south Ethiopia: a community-based cross-sectional study. J Nutr Metab. (2020) 2020(1):5091318. 10.1155/2020/5091318 33062324 PMC7533019

[21] Gebremichael MA Belachew Lema T. Dietary diversity, nutritional status, and associated factors among pregnant women in their first trimester of pregnancy in Ambo district, western Ethiopia. Nutr Metab Insights. (2023) 16:11786388231190515. 10.1177/11786388231190515 38073856 PMC10704939

[22] Shenka A Damena M Abdo M Roba KT. Dietary diversity and nutritional status of pregnant women attending public hospitals in Dire Dawa city administration, eastern Ethiopia. East Afr Health Biomed Sci. (2018) 2(1):10–7.

[23] Abdirahman M Chege P Kobia J. Nutrition knowledge and dietary practices among pregnant adolescents in Mandera county, Kenya. Food Sci Nutr Res. (2019) 2(2):1–8. 10.33425/2641-4295.1018

[24] Joshi PG Joshi GA Jain S Dubey V. Nutritional status of pregnant women reporting at rural health training centre. Int J Reprod Contracept Obstet Gynecol. (2017) 6(9):3846–50. 10.18203/2320-1770.ijrcog20173684

