# Supplementary material for: Maternal dietary diversity and associated factors among pregnant women visiting public health institutions for antenatal care in Addis Ababa, Ethiopia
Source: Front Glob Womens Health. 2026 Apr 2;7:1552532. doi: 10.3389/fgwh.2026.1552532 (PMC13083179; doi:10.3389/fgwh.2026.1552532)
Supplement: Supplementary file 1 [file Appendix1.docx]

#### Questionnaire

Questionnaire ID_______________

**Part** I**. Socio-demographic status of the respondents**

| Sr. no | **Socio-demographic characteristics** | **Items** | **Skip** |
| --- | --- | --- | --- |
| 101. | How old are you? | ______years |  |
| 102. | What is your marital status? | 1.single  2.married  3.separated  4.windowed |  |
| 103. | What is your religion? | 1. Muslim 2. Orthodox 3. Protestant 4. Catholic 5. Other(specify)------ |  |
| 104. | What is your educational level? | 1.unable to read and write  2.able to read and write  3.primary education  4. secondary education  5. collage and above |  |
| 105. | What is your husband educational level? | 1 unable to read and write  2.able to read and write  3.primary education  4. secondary education  5. collage and above |  |
| 106. | What is your occupation? | 1. Housewife 2. Government employees   3. laborer  4. Student  5. Self-employed  6. Others, specify------------ |  |
| 107. | What is your husband occupation? | 1.Government employees  2. Business  3.Laborer  4.Student  5.Self employed  6. Others _________ |  |
| 108. | Where do you live (your Sub city)? | ______________ |  |
| 109. | How much is your family monthly income? | _________Ethiopian birr |  |
| 110. | Family size | _______number. |  |

**Part II: Pregnancy and obstetric related status of the respondents**

| **Sr. no** | **pregnancy and obstetric related status** | **Items** | **Skip** |
| --- | --- | --- | --- |
| 201 | Have you ever got pregnant before? | 1. Yes 2. No | If no skip to 203 |
| 202 | If yes for question no 201 how many times? | _____number. |  |
| 203 | Have you ever give birth before? | 1. Yes 2. No |  |
| 204 | If yes for question no 203, how many times? | _____number. |  |
| 205 | Pregnancy interval | _____number. |  |
| 206 | Have you ever get child death before? | 1. Yes 2. No |  |
| 207 | If yes for question no 206, in what age became died? | 1.with in 1 month  2.1 month-1year  3.> 1year |  |
| 208 | Where did you deliver your last baby? | 1.Home  2.health institution |  |
| 209 | How many live children do you have? | _____number. |  |
| 210 | Do you have miscarriages/abortions before? | 1.Yes  2.No |  |
| 211 | If yes for question no 210, how many times? | _____number. |  |
| 212 | Have you ever sick before during pregnancy? | 1.Yes  2.No |  |
| 213 | Do you have ANC follow up in current pregnancy? | 1.Yes  2.No | If no skip to 216 |
| 214 | If yes for question no 213, how many times? | _____number. |  |
| 215 | When do you start ANC follow up? | 1. 1^st^ trimester 2. 2^nd^ trimester 3. 3^rd^ trimester |  |
| 216 | What is the stage of your current pregnancy | 1.1^st^ trimester 2.2^nd^ trimester 3.3^rd^ trimester |  |

Part III: questions assessing nutritional practice of the respondents

| Sr. no | Nutritional practice | Items | Skip |
| --- | --- | --- | --- |
| 301. | Do you crave food not normally consumed? | 1. Yes 2. No |  |
| 302 | Do you avoid any food items during current pregnancy? | 1. Yes 2. No | If no skip to 304 |
| 303. | If yes for Q.302 what is your reason? | 1.Personal dislike  2.Religion  3.Makes fetus big  4.Culture  5.Other |  |
| 304. | Do you Follow specific dietary regimen? | 1. Yes 2. No |  |
| 305. | How many times do you eat per day? | __________number |  |
| 306. | Do you have a habit of eating snack? | 1. Yes 2. No |  |
| 307. | Do you skip any meal during current pregnancy? | 1. Yes 2. No |  |
| 308. | If yes for Q. 307 which meal do you skip? | 1.Breakfast  2.Lunch  3.Dinner |  |
| 309 | Do you eat additional food? | 1. Yes 2. No |  |
| 310 | If yes for Q. 309 how many times you eat additional meal per day? | _________number |  |
| 311 | Do you eat protein rich foods during current pregnancy? | 1. Yes 2. No |  |
| 312 | Do you have a habit of eating fresh fruits and vegetable? | 1. Yes 2. No |  |
| 313. | Which type of salt you use in your food? | 1.iodized salt  2.not iodized salt |  |
| 314 | When you add salt in cooking food? | 1.At the end of cooking  2.At the middle of cooking  3.At the beginning of cooking  4.Cook without any salt |  |
| 315 | How much fluid do you drink per day? | ______litter. |  |
| 316 | Do you drink coffee or tea? | 1. Yes 2. No |  |
| 317 | If yes for Q.316 how much you drink per day? | _________cups. |  |
| 318 | Do you have iron supplement during current pregnancy? | 1. Yes 2. No |  |
| 319 | Did you drink alcohol? | 1. Yes 2. No |  |

**Part V. Food group category of 24 hr recall of pregnant women nutritional consumption practice**

| Number | Food group | Examples | 1. Yes 2. No |
| --- | --- | --- | --- |
|  | Cereals ( grains, white roots and tubers an plantain | com/maize, rice, wheat, sorghum, teff, millet or any other grains or foods made from these (e.g. biscuits, pasta, Macaroni, bread, noodles, porridge or other grain products) + insert local foods e.g Enjera,kocho, chechepsa, white potatoes, white yam, white cassava, or other foods made from roots |  |
|  | Pulses ( beans, peas and lentils) | Dried beans, dried peas and dried lentils |  |
|  | Nuts and seeds | Nuts, seeds of foods made from these ( e.g. peanut butter) |  |
|  | Dairy products | Milk, cheese yoghurt, butter or other milk products |  |
|  | Meat, poultry and fish | Beef, pork, sheep, goat, chicken and others meat, Omega, fresh or dried fish or shellfish. |  |
|  | Eggs | Eggs from chiken |  |
|  | Dark green leafy vegetables | dark green leafy vegetables, including wild forms + locally available vitamin A rich leaves such as Salad(lettuce), kale, spinach(cabbage) |  |
|  | Other vitamin A rich fruits and vegetables. | Pumpkin(duba), carrot, sweet potato that are orange inside + other locally available vitamin A rich vegetables (e.g.red sweet pepper), ripe mango, apple, avocado, melon(habab), lemon, apricot (fresh or dried), strawberry, ripe papaya, dried peach, and 100% fruit juice made from these + other locally available vitamin A rich fruit |  |
|  | Other vegetable | other vegetables (e.g. tomato, onion, eggplant) + other locally available vegetables |  |
|  | Other fruits | other fruits, including wild fruits and 100% fruit juice made from these |  |
